# Regulatory effects of a novel cysteine protease inhibitor in *Baylisascaris schroederi* migratory larvae on mice immune cells

**DOI:** 10.1186/s13071-022-05240-8

**Published:** 2022-04-04

**Authors:** Jing-Yun Xu, XiaoBin Gu, Yue Xie, Ran He, Jing Xu, Lang Xiong, XueRong Peng, GuangYou Yang

**Affiliations:** 1 Department of Parasitology, College of Veterinary Medicine, Sichuan Agricultural University, Wenjiang, 611130 People’s Republic of China; 2 Department of Chemistry, College of Life and Basic Science, Sichuan Agricultural University, Wenjiang, 611130 People’s Republic of China

**Keywords:** *Baylisascaris schroederi*, Cysteine protease inhibitor, PBMC, TLRs signal pathway, Immune evasion mechanism

## Abstract

**Background:**

The giant panda (*Ailuropoda melanoleuca*) is a well-known, rare and endangered species. *Baylisascaris schroederi* is a pathogenic ascarid. Infection with *B. schroederi* may cause death in giant pandas. At present, the immune evasion mechanism of *B. schroederi* is little known. Cysteine protease inhibitors (CPI) play important roles in the regulation of host immune responses against certain nematodes. In this study, we focused on the analysis of the regulation of *B. schroederi* migratory larvae CPI (rBsCPI-1) on mice immune cells.

**Methods:**

First, the pattern recognition receptors on the surface of peripheral blood mononuclear cells (PBMCs) and the signal pathways that transduce extracellular signals into the nucleus activated by rBsCPI-1 were identified. Then, the regulatory effects of rBsCPI-1 on PBMCs physiological activities were detected. Finally, the effects of rBsCPI-1 on TLR signaling pathway activation and NF-κB phosphorylation in mice immunized with recombinant protein were analysed.

**Results:**

The results suggested that rBsCPI-1 secreted by *B. schroederi* migratory larvae is mainly recognized by TLR2 and TLR4 on PBMCs. Extracellular signals are transduced into the nucleus through the MAPK and NF-κB signaling pathways, enhancing the phagocytosis, migration, and apoptosis of PBMCs; meanwhile, rBsCPI-1 induces high expression of NO. Thus, rBsCPI-1 plays a role in immune regulation. In addition, the high expression of negative regulatory factors also ensured that TLR activation is maintained at the optimal level.

**Conclusions:**

rBsCPI-1 can transduce regulatory signals into immune cells by activating the TLR2/4-NF-κB/MAPK signaling pathway, having a certain regulatory effect on the physiological activities. Meanwhile, rBsCPI-1 can maintain the immune response in a balance by limiting the over-activation of the TLRs signaling pathway and thus contributes to *B. schroederi* immune evasion.

**Graphical Abstract:**

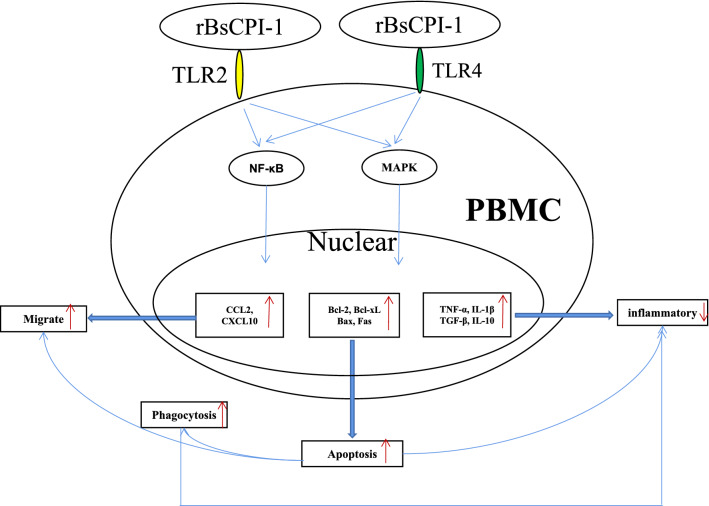

**Supplementary Information:**

The online version contains supplementary material available at 10.1186/s13071-022-05240-8.

## Background

The giant panda (*Ailuropoda melanoleuca*), a well-known, rare and endangered species, is a powerful symbol of species conservation. Owing to their highly simplistic bamboo diet, various serious infectious diseases threaten the species [1]. Among them, parasitic infection seems to be one of the crucial factors bringing its population on the verge of extinction [2, 3]. As the obligate host of *Baylisascaris schroederi* (*B. schroederi*), giant pandas in various nature reserves and artificial breeding centers in China are commonly found to be infected with *B. schroederi* [4]. Although previous studies have experimentally investigated *B. schroederi* biology, population genetic structure, genome and mitochondrial genome, diagnostic methods, and other aspects [5–7], its immune evasion mechanism remains little known.

Parasitic nematodes are continuously attacked by the host immune system. To survive in the host for long periods, they have to develop effective immune evasion strategies [8]. Nematodes belonging to the *Ascaris* spp. (e.g., *A. lumbricoides* and *A. suum*) and *Baylisascaris* spp. (e.g., *B. transfuga*, *B. procyonis*, *B. ailuri*, and *B. schroederi*) complete their life cycle in the host; the larvae undergo complex migration and development processes. The larvae migrate through the liver and lung and then return to the small intestine to develop into adults. *B. schroederi* migration larvae can migrate to various organs of giant pandas and induce "visceral larva migrans (VLM)", causing severe intestinal injury, helminthic hepatitis and pneumonia, and greatly affect their survival and health [9]. At present, research on the immune regulation mechanism of *B. schroederi* during migration is lacking.

Cysteine protease inhibitor (CPI) belongs to the superfamily of reversible, papain-like cysteine protease inhibitors and is widely found in plants, animals, and microorganisms [10]. Functionally, CPI is involved in various physiological and cellular processes, including immune and inflammatory responses, protein homeostasis, cell–matrix remodeling, and apoptosis [11]. Moreover, CPI plays an essential role in the regulation of immune modulators secreted by some nematodes [12, 13]. We previously found some CPIs through the structural domain, GO and KEGG functional annotation of *B. schroederi* secreting proteins [7], and speculated that *B. schroederi* CPIs may play important roles in host invasion, tissue degradation, immune regulation and evasion. However, it is unclear whether its specific mechanism is similar to other nematodes.

In this study, we observed the immunomodulatory effects of rBsCPI-1, which is highly expressed in *B. schroederi* migratory larvae, on mice peripheral blood mononuclear cells (PBMCs). Moreover, we analyzed its dual regulation of TLR signaling pathway. These results will provide a basis for understanding the biological and immunological functions of this protein in host-parasite interactions.

## Methods

### Experimental animals and cells

Six-eight-week-old, specific pathogen-free (SPF), female BALB/c mice (n = 40) were purchased from Chengdu Dashuo Laboratory Animal Co., Ltd. The animal study protocol was reviewed and approved by the Animal Care and Use Committee of Sichuan Agricultural University (SYXK 2019–189). All animal procedures used in this study were performed in accordance with the Guide for the Care and Use of Laboratory Animals (National Research Council, Bethesda, MD, USA) and recommendations of the Animal Research: Reporting of In Vivo Experiments (ARRIVE) guidelines (http://www.nc3rs.org.uk/arrive-guidelines). All applicable institutional and national guidelines for the care and use of animals were followed.

Blood samples were taken from six mice at one time and used as independent samples for subsequent experiments. PBMCs were separated using the Mouse Peripheral Blood Mononuclear Cell Separation Solution KIT (TBD, China) according to manufacturer’s instructions. PBMCs (1 × 10^6^/mL) were cultured with pre-warmed (37 °C) RPMI 1640 (Hyclone, USA) complete medium with 10% fetal bovine serum (FBS; Sigma, USA), 100 U/mL penicillin, and 100 μg/mL streptomycin (Solarbio, China) at 37 °C and 5% CO_2_.

### Preparation of recombinant *B. schroederi* CPI (rBsCPI-1)

The preparation of rBsCPI-1 as previous (Xu et al.). The BsCPI-1 (accession number: OM780049) expressed at a high level in *B. schroederi* migratory larvae was identified by quantitative real-time PCR (qRT-PCR). The amplified fragments of the BsCPI-1 gene were successfully obtained by PCR from *B. schroederi* migratory larvae cDNA with specific pair of primers. The primer sequences containing the BamHI and EcoRI restriction sites (bold) were 5ʹ-CGC**GGATCC**ATGCGCGCGGCAATGC-3ʹ and 5ʹ-CCG**GAATTC**TTAAGAGGTCTCCTTGATTTCTTTTATGGT-3ʹ. Then successfully constructed the recombinant pET-32a(+)-BsCPI-1 plasmid. The isopropyl-β-D-thiogalactopyranoside (IPTG) (Sigma, USA) induced protein product of BsCPI-1 expressed in *Escherichia coli* (*E. coli*) (BL21) cells (Takara, Japan). The pET-32a-BsCPI-1 positive expression bacterial solution was expressed. Then, protein purification was performed using a nickel (Ni) column. The concentration of the rBsCPI-1 protein was determined by bicinchoninic acid (BCA) assay (Takarabio, China). Contaminated endotoxin was removed by ToxOut™ High-Capacity Endotoxin Removal Kit (GenScript, China). At the same time, the endotoxin contented in rBsCPI-1detected by the ToxinSensor™ Chromogenic LAL Endotoxin Assay Kit (GenScript) was less than 0.1 EU/mL, it can be considered successfully removed. An analysis of enzyme inhibitory activity [14] of rBsCPI-1 showed that it effectively inhibited cathepsin L, cathepsin B, and papain in a dose-dependent manner (Xu et al.).

### Binding of rBsCPI-1 with PBMC proteins

PBMCs growing in suspension were collected by centrifuging at 2000 r/m for 10 min and resuspended in RIPA lysis buffer and 1% PMSF (Solarbio, China) to lyse the suspension cells; Meanwhile, RIPA lysis buffer and 1% PMSF were added to each well to lyse the adherent cells. After lysis was complete, the samples were centrifuged at 12,000×*g* for 5 min, and the PBMC protein concentration was examined using the BCA assay kit.

Six mice were intraperitoneally immunized with 50 μg rBsCPI-1. Booster immunization was administered two times with 50 μg rBsCPI-1 with 7 days intervals. Tail blood from immunized mice was collected 7 days after the final immunization, and anti-rBsCPI-1 serum was isolated. Six mice were orally infected with 5000 infectious eggs (the second-stage larvae (L2) in eggs). Tail blood from infected mice was collected 24 h after the infection, and infection serum was isolated.

Binding of rBsCPI-1 with PBMC proteins was assessed using ELISA. In brief, the ELISA plates were coated with 100 µL PBMC proteins at different concentrations (0.5, 1, 2, 4, 6, 8, and 10 µg/mL) and incubated at 4 °C overnight. After blocking with 5% skim milk and washing thrice, 100 µL rBsCPI-1 at different concentrations (0.5, 1, 5, 10, 15, 20, and 25 µg/mL) was added to the wells, and the mixture was incubated at 37 °C for 2 h. After washing, anti rBsCPI-1 serum (1:100) and horseradish peroxidase (HRP)-conjugated rabbit anti-mouse IgG (1:10,000; Sigma, USA) were added. Absorbance values at 492 nm were measured.

### Cell proliferation assay

Approximately 100 µL PBMC suspension (1 × 10^6^/mL) were treated with Concanavalin A (ConA: 10 µg/mL) alone or in the presence of different concentrations of rBsCPI-1 (0, 1, 5, 10, 15, 25, and 50 µg/mL), respectively, for different incubation periods (0, 2, 4, 6, 12, 18, and 24 h). Next, 10 µL CCK-8 solution (meilunbio, Dalian, China) was added to each well and incubated for 4 h before harvesting, and the absorbance values were measured at 450 nm.

### qRT-PCR of TLR and NLR genes

Approximately 100 µL PBMC suspension (1 × 10^6^/mL) were treated with phosphate-buffered saline (PBS), pET-32a, and rBsCPI-1. Total RNA was extracted from PBMCs using the Total RNA Extraction Kit (Solarbio, China). cDNA was synthesized from total RNA using the PrimeScript 1st Strand cDNA Synthesis Kit (Takara, Japan). The relative expression of TLR and NLR genes in PBMCs was evaluated using qRT-PCR. Additional file 1: Table S1 shows the primer sequences for each investigated gene. qRT-PCR reactions were performed using Roche LightCycler 96. Amplifications were conducted with a 20 μL reaction volume containing 10 μL of TB Green Premix Ex Taq™ (Tli RNase H Plus) (TaKaRa, Japan), 2 μL of cDNA template (50 ng), 0.8 μL of forward and reverse primers (10 μM), and 6.4 μL of ddH_2_O. The PCR amplification procedure was as follows: 95 °C for 10 min; 40 cycles of 95 °C for 5 s, 60 °C for 30 s; and 95 °C for 5 s, 60 °C for 60 s, 95 °C for 1 s. Screened GAPDH among three housekeeping genes (18 s, GAPDH, β-actin) by pre-experiment as the experimental housekeeping control. Transcription levels of the target genes were normalized by subtracting the expression level of GAPDH and then calculating the relative expression using the 2^−∆∆Ct^ method.

### Western blotting analysis of the activation of NF-κB and MAPK signaling pathways

The preparation method of PBMC proteins was processed as mentioned above. A total of 50 µg PBMC proteins was separated using SDS-PAGE. The proteins were blotted onto nitrocellulose filter membranes. The membranes were placed into 5% skim milk for 2 h at room temperature. Subsequently, the membranes were incubated with anti-NF-κB (1:2000), anti-p38 MAPK (1:2000), anti-ERK1/2 (1:2000), anti-JNK1/2 (1:1000), anti-p-NF-κB (1:1000), anti-p-p38 MAPK (1:2000), anti-p-ERK1/2 (1:2000), and anti-p-JNK1/2 (1:2000; diluted in 5% skim milk, ABclone), respectively, at 4 °C overnight. After washing three times, the membranes were incubated with HRP-conjugated secondary antibody (1:5000; diluted in 5% skim milk, ABclone) for 2 h at room temperature. After washing, the membranes were exposed using ultrasensitive ECL chemiluminescence reagent (Meilunbio, China). The bands were quantified using densitometry and analyzed with Image J.

### qRT-PCR analysis of negative regulators in the TLR pathway

The relative expression of negative regulators in the TLR pathway (Interleukin 1 Receptor Associated Kinase 2, IRAK-2; Interleukin 1 Receptor Associated Kinase M, IRAK-M; Suppressor of Cytokine Signaling, SOCS; Toll Interacting Protein, Tollip; Tripartite Motif Protein 30α, TRIM-30α; Zinc Finger Protein A20, A20; and Single Immunoglobulin IL-1-Related Receptor, SIGIRR) was determined on the same qRT-PCR run as mentioned above. Additional file 1: Table S1 shows the primer sequences for each investigated gene.

### Cell apoptosis assay

The apoptosis assay was performed according to the instructions of Annexin V-FITC/PI Apoptosis Assay Kit (Vazyme, China). Approximately 100 µL PBMC suspension (1 × 10^6^/mL) were treated with PBS (control), pET-32a, and rBsCPI-1, respectively, then centrifuged at 2 000 r/m for 10 min. Resuspended the cells in 100 μL 1 × Binding Buffer and added 5 µL Annexin V-FITC with 5 µL PI Staining Solution. Then incubated in the dark at room temperature for 10 min. Added 400 μL 1 × Binding Buffer and analyzed immediately by flow cytometry (FCM) (BD Biosciences, San Jose, CA, USA).

The relative expression of pro-apoptotic and anti-apoptotic genes was estimated on the same qRT-PCR run as mentioned above. Additional file 1: Table S1 shows the primer sequences for each investigated gene.

### Cell phagocytosis assay

In brief, approximately 100 µL PBMC suspension (1 × 10^6^/mL) were treated with PBS (control), pET-32a, and rBsCPI-1, respectively, then centrifuged at 2 000 r/m for 10 min. The cells were resuspended in 1 mL FITC-dextran (Sigma, USA) in RPMI 1640 (1 mg/mL) and incubated in the dark at 37 °C for 1 h. 1 mL pre-cooled PBS containing 2% FBS was added to stop the reaction. The cells were washed three times with PBS and resuspended in 500 μL PBS containing 2% paraformaldehyde for 10 min, then analyzed the internalization of FITC-dextran by PBMCs immediately using FCM.

### Analysis of cytokine expression

The relative expression of transforming growth factor β (TGF-β), interleukin 10 (IL-10), tumor necrosis factor α (TNF-α) and IL-1β in PBMCs were evaluated on the same qRT-PCR run as mentioned above.

### Cell migration assay

The cell migration assay was performed using a Millicell® insert with 8 µm pores (Merck Millipore, Germany) according to the manufacturer’s instructions. PBMCs (1 × 10^6^/mL) were incubated with PBS (control), pET-32a, and rBsCPI-1, respectively, at 37 °C and 5% CO_2_. The cells (200 µL) were seeded into the upper chamber, and the lower chamber was filled with 1300 µL RPMI 1640 complement medium. Then, the cells that migrated through the polycarbonate membrane into the lower chamber were counted using a Neubauer counting chamber.

The relative expression of chemokines was measured on the same qRT-PCR run as mentioned above. Additional file 1: Table S1 shows the primer sequences for each investigated gene.

### NO production assay

PBMCs (100 µL; 1 × 10^6^/mL) were incubated with PBS (control), pET-32a, and rBsCPI-1, respectively, at 37 °C and 5% CO_2_, and intracellular NO production was determined using the Total Nitric Oxide Assay Kit (Solarbio, China) according to the instructions. Absorbance was measured at 540 nm, and NO production in µmol/L was estimated using a standard curve.

### In vivo experiment

In total, 18 mice were intraperitoneally injected with 50 µg pET-32a, rBsCPI-1, and equal volume of PBS three times at 7 days interval, respectively. Then, the mice were sacrificed 7 days after the last immunization, and PBMCs were collected. The relative expression of TLR2, TLR4, and negative regulators of the TLR pathway were measured on the same qRT-PCR run as mentioned above.

### Statistical analysis

All data are expressed as mean ± standard deviation (SD). Statistical analysis was done using GraphPad Prism 5. Image J software was used to quantify the protein band intensity. Differences between groups were assessed by one-way analysis of variance (ANOVA) in SPSS 11.5. *P* < 0.05 was considered to indicate statistical significance.

## Results

### Peripheral blood mononuclear cells (PBMCs) recognize and bind rBsCPI-1 through Toll-like receptors (TLRs)

#### Binding of rBsCPI-1 to PBMCs

**Fig. 1:**
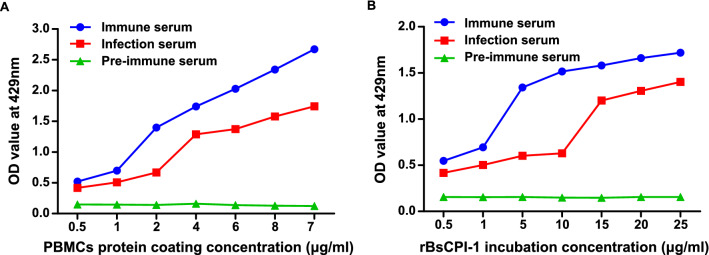
Binding of recombinant cysteine protease inhibitor of *Baylisascaris schroederi* migratory larvae (rBsCPI-1) with peripheral blood mononuclear cells (PBMCs) as detected by enzyme-linked immunosorbent assay (ELISA). **A** Binding of PBMC proteins at various coating concentrations when incubated with 1 µg/mL rBsCPI-1. **B** Binding of 1 µg/mL PBMC proteins when incubated with different concentrations of rBsCPI-1. Data are shown as mean ± SD of 3 replicates per group

As shown in Fig. 1, with the increase in PBMC protein coating concentration, the optical density (OD) value of PBMC proteins bound to rBsCPI-1 showed an increasing trend. In addition, as the coating concentration of rBsCPI-1 increased, the OD value of PBMC proteins bound to rBsCPI-1 also showed an increasing trend. This finding indicates that rBsCPI-1 binds PBMC proteins and there is a significant interaction between them.

#### Effects of rBsCPI-1 on PBMC proliferation

**Fig. 2:**
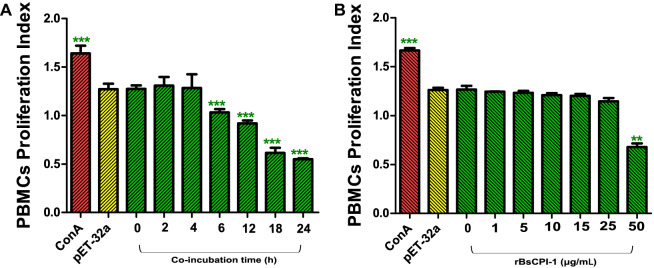
Recombinant cysteine protease inhibitor of *Baylisascaris schroederi* migratory larvae (rBsCPI-1) affects the proliferation of peripheral blood mononuclear cells (PBMCs). Proliferation of PBMCs treated with ConA along or combined with rBsCPI-1 were measured by Cell Counting Kit 8 (CCK-8) assay. The OD_450_ values were considered as the cell proliferation index. PBMCs used for all replicates of distinct treatments in each experimental repetition were derived from the same mouse. Data are shown as mean ± SD of 3 replicates per group. ^*^P < 0.05, ^**^P < 0.01, ^***^P < 0.001 for other groups vs 0 µg/mL group; or other groups vs 0 h group

The Cell Counting Kit 8 (CCK-8) assay was used to detect the effect of rBsCPI-1 on the proliferation of PBMCs and to determine the optimal reaction concentration and co-incubation time for subsequent experiments. As seen in Fig. 2B, compared with 0 μg/mL CPI-1 stimulation group, the proliferation of PBMCs was gradually inhibited with the increase in protein concentration, and it decreased significantly when the concentration of rBsCPI-1 reached 50 µg/mL (*P* < 0.001). Moreover, the proliferation of PBMCs also decreased gradually with increasing co-incubation time, and it was significantly inhibited when the co-incubation time was 6 h (*P* < 0.05; Fig. 2A). Therefore, to exclude the influence of cell proliferation on subsequent experiments, 25 µg/mL and 4 h were selected as the optimal reaction concentration and co-incubation time, respectively, for rBsCPI-1.

#### PBMCs recognize rBsCPI-1 through TLR2 and TLR4

**Fig. 3:**
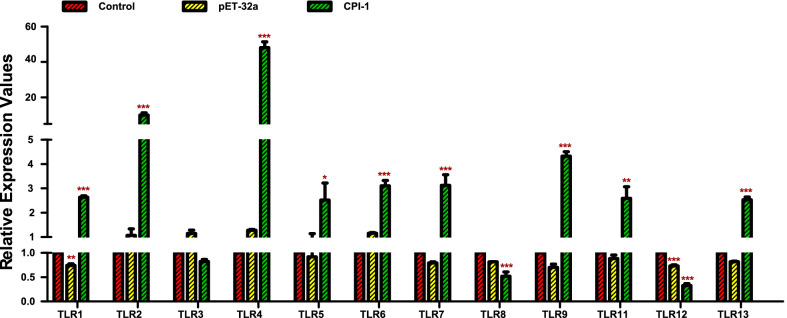
Recombinant cysteine protease inhibitor of *Baylisascaris schroederi* migratory larvae (rBsCPI-1) affects the relative expression of Toll-like receptor (*Tlr*) genes in peripheral blood mononuclear cells (PBMCs). Data are shown as mean ± SD of 3 replicates per group. ^*^P < 0.05, ^**^P < 0.01, ^***^P < 0.001 versus control group

To analyze the specific mechanism underlying the immunomodulatory role of PBMCs, we further detected the transcription levels of genes belonging to the TLR and Nod-like receptor (NLR) families. qRT-PCR results showed that the transcription levels of TLR1, TLR2, TLR4, TLR5, TLR6, TLR7, TLR9, TLR11, and TLR13 were significantly higher in the presence of rBsCPI-1, and the changes in the transcription levels of TLR2 and TLR4 were the most significant (Fig. 3). Moreover, the transcription levels of NLRP3 and NLRP6 were also significantly higher in the presence of rBsCPI-1 (*P* < 0.001; Additional file 2: Fig. S1).

#### rBsCPI-1 activates downstream signaling pathways of TLRs

**Fig. 4:**
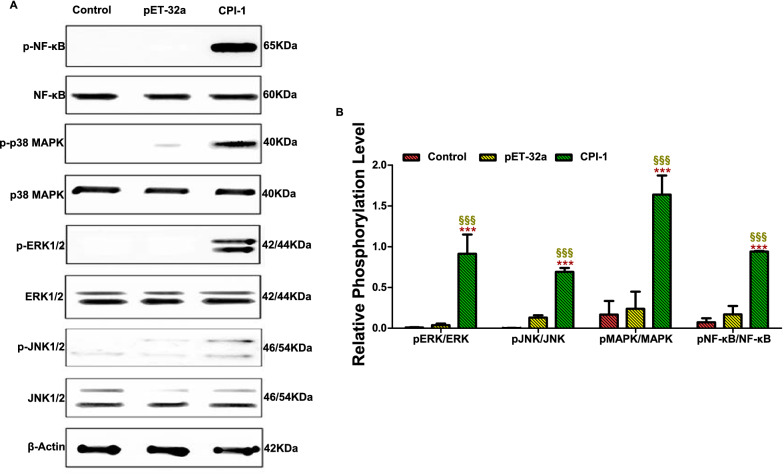
Western blotting detected the activation of the MAPK and NF-κB signaling pathways. A representative Western blotting is shown in **A**, and the graph of the quantified band density is shown in **B**. Data are shown as mean ± SD of 3 replicates per group. ^*^P < 0.05, ^**^P < 0.01, ^***^P < 0.001 versus control group; ^§^*P* < 0.05, ^§§^*P* < 0.01, ^§§§^*P* < 0.001 versus pET-32a group

Western blotting was used to detect the phosphorylation levels of NF-κB, p38 MAPK, JNK1/2, and ERK1/2 proteins to further analyze the effect of rBsCPI-1 on the activation of downstream signal transduction pathways of TLRs. The gray values of the protein bands were quantified using Image J (Fig. 4). Compared with the control group, rBsCPI-1 induced a significant increase in the phosphorylation levels of NF-κB, p38 MAPK, JNK1/2, and ERK1/2 proteins (*P* < 0.001), and there was no significant difference between the pET-32a group and the control group (*P* > 0.05). Therefore, we speculated that rBsCPI-1 is recognized by TLRs on the surface of PBMCs, and it participates in inflammation or regulates cell physiological activities by inducing the phosphorylation of proteins in the downstream signal transduction pathways.

### rBsCPI-1 activates the negative feedback pathway of TLRs

**Fig. 5:**
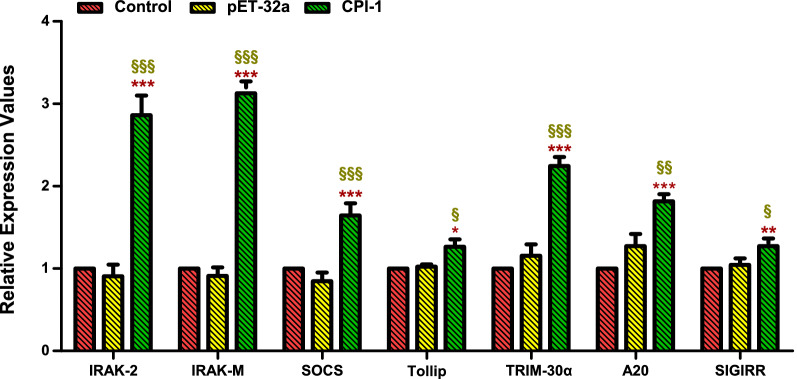
Quantitative real-time PCR (qRT-PCR) detected the relative expression of the negative regulators of the Toll-like receptor (TLR) signaling pathway. Data are shown as mean ± SD of 3 replicates per group. ^*^P < 0.05, ^**^P < 0.01, ^***^P < 0.001 versus control group; ^§^*P* < 0.05, ^§§^*P* < 0.01, ^§§§^*P* < 0.001 versus pET-32a group

The qRT-PCR results showed that rBsCPI-1 significantly increased the transcription levels of the negative regulators (IRAK-2, IRAK-M, SOCS, Tollip, TRIM-30α, A20, and SIGIRR) in the TLR signaling pathway. Compared with the control group, pET-32a did not cause significant changes in the transcription levels of these negative regulators (Fig. 5). Thus, rBsCPI-1 may play a bidirectional regulatory role in the TLR signaling pathway.

#### Effects of rBsCPI-1 on apoptosis of PBMCs

**Fig. 6:**
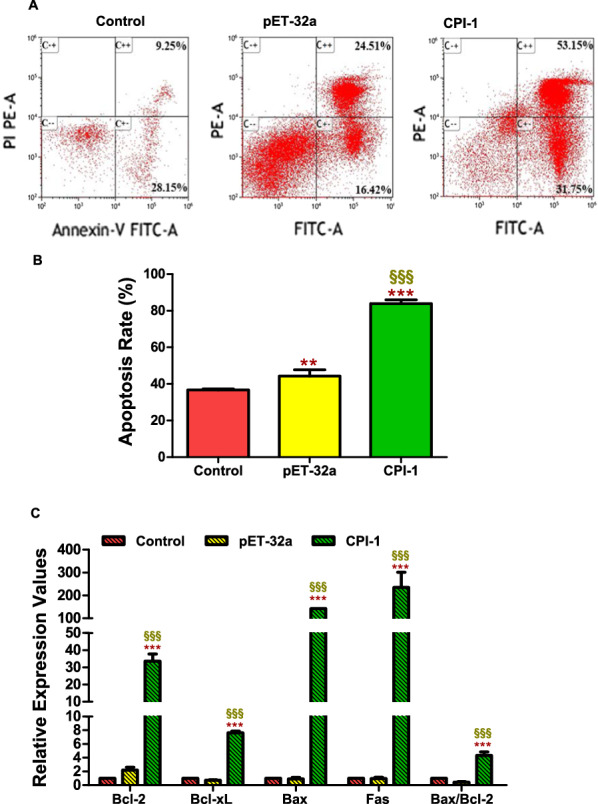
Recombinant cysteine protease inhibitor of *Baylisascaris schroederi* migratory larvae (rBsCPI-1) affects the apoptosis of peripheral blood mononuclear cells (PBMCs). Apoptosis of PBMCs was determined by staining with annexin V and PI followed by flow cytometry (**A**). The total apoptosis rate presented is representative of three independent experiments (**B**). qRT-PCR detected the transcription levels of pro-apoptotic genes (*Fas*, *Bax*) and anti-apoptotic genes (*Bcl-2*, *Bcl-xL*) (**C**). Data are presented as mean ± SD. ^*^P < 0.05, ^**^P < 0.01, ^***^P < 0.001 versus control group; ^§^*P* < 0.05, ^§§^*P* < 0.01, ^§§§^*P* < 0.001 versus pET-32a group

Apoptosis of PBMCs was determined by staining with Annexin V and PI followed by FCM. AnnexinV FITC + /PI PE + cells indicated late apoptosis cells and AnnexinV FITC + /PI PE- cells indicated early apoptosis cells. rBsCPI-1 significantly increased the total apoptosis rate (early apoptosis rate + late apoptosis rate) of PBMCs compared with the control group (*P* < 0.001). pET-32a also promoted the total apoptosis rate (*P* < 0.01), but the ability of rBsCPI-1 to induce apoptosis was significantly higher than that of pET-32a (*P* < 0.001; Fig. 6B).

We used qRT-PCR to analyze the transcription levels of the pro-apoptotic factors (Bax and Fas) and anti-apoptotic factors (Bcl-2 and Bcl-xL). The results showed that compared with the control group, rBsCPI-1 significantly increased the transcription levels of the pro-apoptotic and anti-apoptotic genes in PBMCs. The ratio of Bax/Bcl-2 transcription levels also increased significantly in the rBsCPI-1 group (Fig. 6C). Therefore, we speculated that rBsCPI-1 has a dual regulatory effect on the apoptosis of PBMCs, but the pro-apoptotic effect was dominant.

#### Effects of rBsCPI-1 on phagocytosis of PBMCs

**Fig. 7:**
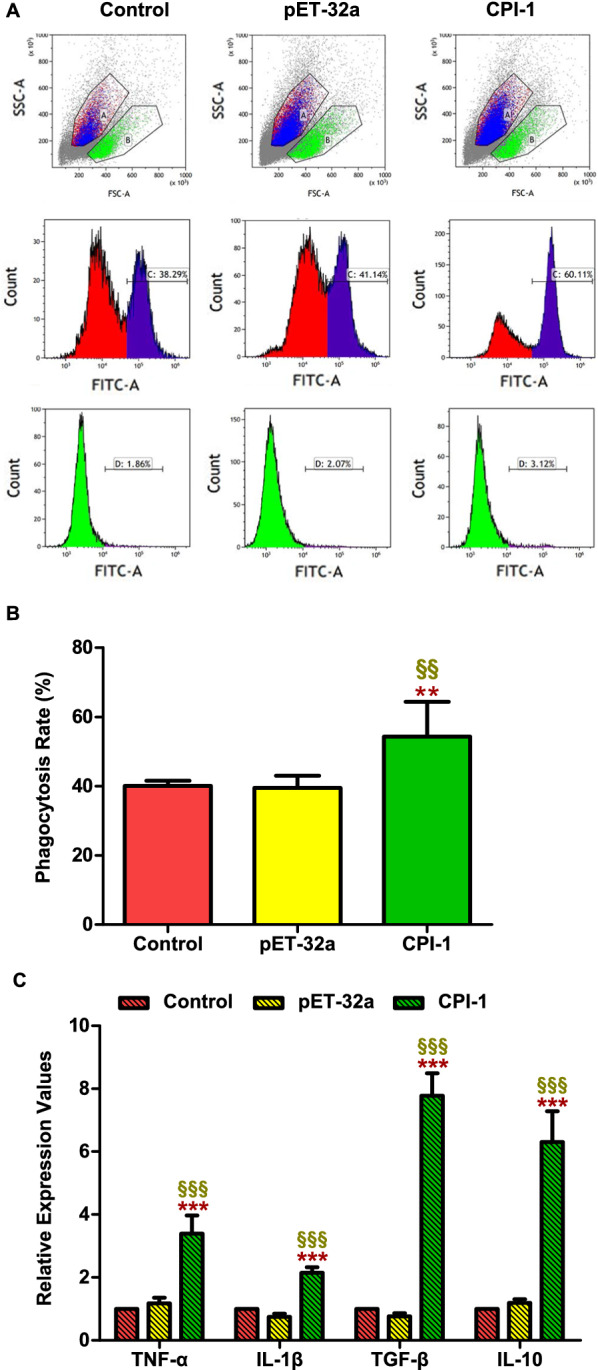
Recombinant cysteine protease inhibitor of *Baylisascaris schroederi* migratory larvae (rBsCPI-1) increased the phagocytosis of peripheral blood mononuclear cells (PBMCs). Phagocytosis of PBMCs was determined by phagocytosed FITC-dextran using flow cytometry (**A**). The percentage of FITC^+^ cells presented is representative of three independent experiments (**B**). The relative expression of pro-inflammatory and anti-inflammatory cytokines is shown in (**C**). Data are shown as mean ± SD of 3 replicates per group. ^*^P < 0.05, ^**^P < 0.01, ^***^P < 0.001 versus control group; ^§^*P* < 0.05, ^§§^*P* < 0.01, ^§§§^*P* < 0.001 versus pET-32a group

The cell phagocytosis assay was performed by FCM to explore the effects of rBsCPI-1 on phagocytosis of PBMCs, and the phagocytosis index = FITC + cells/the total count cells (Fig. 7A). The results showed that rBsCPI-1 significantly increased the phagocytosis of PBMCs (*P* < 0.01) compared with the control group, meanwhile, pET-32a (*P* > 0.05) had no marked regulatory effect on the phagocytosis of PBMCs (Fig. 7B).

### Detection of the cytokine levels

In addition, we detected the changes in the expression of anti-inflammatory factors TGF-β and IL-10 and pro-inflammatory factors TNF-α and IL-1β by qRT-PCR. The results showed that rBsCPI-1 induced a higher expression of TGF-β, IL-10, TNF-α, and IL-1β compared with the control and pET-32a group, but the change levels of pro-inflammatory factors expression were lower than those of anti-inflammatory factors (Fig. 7C). Therefore, we speculated that rBsCPI-1 has a dual regulatory effect on the induction of cytokines, and the effect of inducing the expression of anti-inflammatory factors was more significant.

#### Effects of rBsCPI-1 on migration of PBMCs

**Fig. 8:**
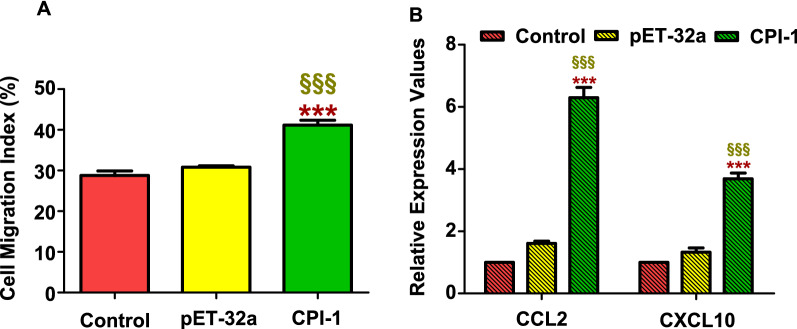
Effects of recombinant cysteine protease inhibitor of *Baylisascaris schroederi* migratory larvae (rBsCPI-1) on peripheral blood mononuclear cell (PBMC) migration. Cells were incubated with PBS (control), pET-32a, and rBsCPI-1. Then, the random migration was determined using a Neubauer counting chamber; the migration index was calculated as number of cells in the lower chamber/2 × 10^5^ cells (**A**). The relative expression of chemokines (*Ccl2*, *Cxcl10*) is shown in **B**. Data are shown as mean ± SD of 3 replicates per group. ^*^P < 0.05, ^**^P < 0.01, ^***^P < 0.001 versus control group; ^§^*P* < 0.05, ^§§^*P* < 0.01, ^§§§^*P* < 0.001 versus pET-32a group

The cell migration assay was performed to analyze whether the migration of PBMCs was significantly affected by rBsCPI-1. The experimental results showed that rBsCPI-1 significantly promoted the migration of PBMCs compared with the control group (*P* < 0.001; Fig. 8A). Therefore, we speculated that rBsCPI-1 facilitates the migration of PBMCs to the site of infection.

At the same time, we also detected the transcriptional levels of chemokines CCL2 and CXCL10 by qRT-PCR. The transcription levels of CCL2 and CXCL10 were significantly higher in the rBsCPI-1 group than those in the control and pET-32a group (*P* < 0.001; Fig. 8B). Therefore, we speculated that rBsCPI-1 can induce directional migration of immune cells by inducing high expression of chemokines.

#### Effects of rBsCPI-1 on NO production

**Fig. 9:**
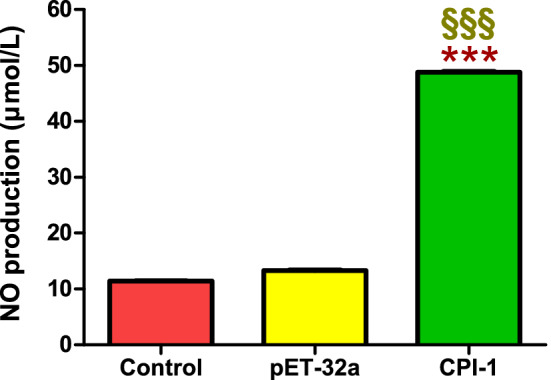
Effects of recombinant cysteine protease inhibitor of *Baylisascaris schroederi* migratory larvae (rBsCPI-1) on NO production by peripheral blood mononuclear cells (PBMCs). The NO concentration (µmol/L) was calculated using a standard curve. Data are shown as mean ± SD of 3 replicates per group. ^*^P < 0.05, ^**^P < 0.01, ^***^P < 0.001 versus control group; ^§^*P* < 0.05, ^§§^*P* < 0.01, ^§§§^*P* < 0.001 versus pET-32a group

NO is involved in most parasitic infections and mediates non-specific host defense by eliminating parasites or delaying parasite growth. The Total Nitric Oxide Assay Kit was used to determine whether rBsCPI-1 also induced PBMCs to produce NO. The results showed that compared with the control group, the NO production level of the pET-32a group did not differ significantly (*P* > 0.05). In contrast, the NO production level in the rBsCPI-1 group was significantly higher (*P* < 0.001; Fig. 9). Therefore, we speculated that rBsCPI-1 mediates the elimination of *B. schroederi* by inducing PBMCs to produce a high level of NO.

### In vivo experiment

**Fig. 10:**
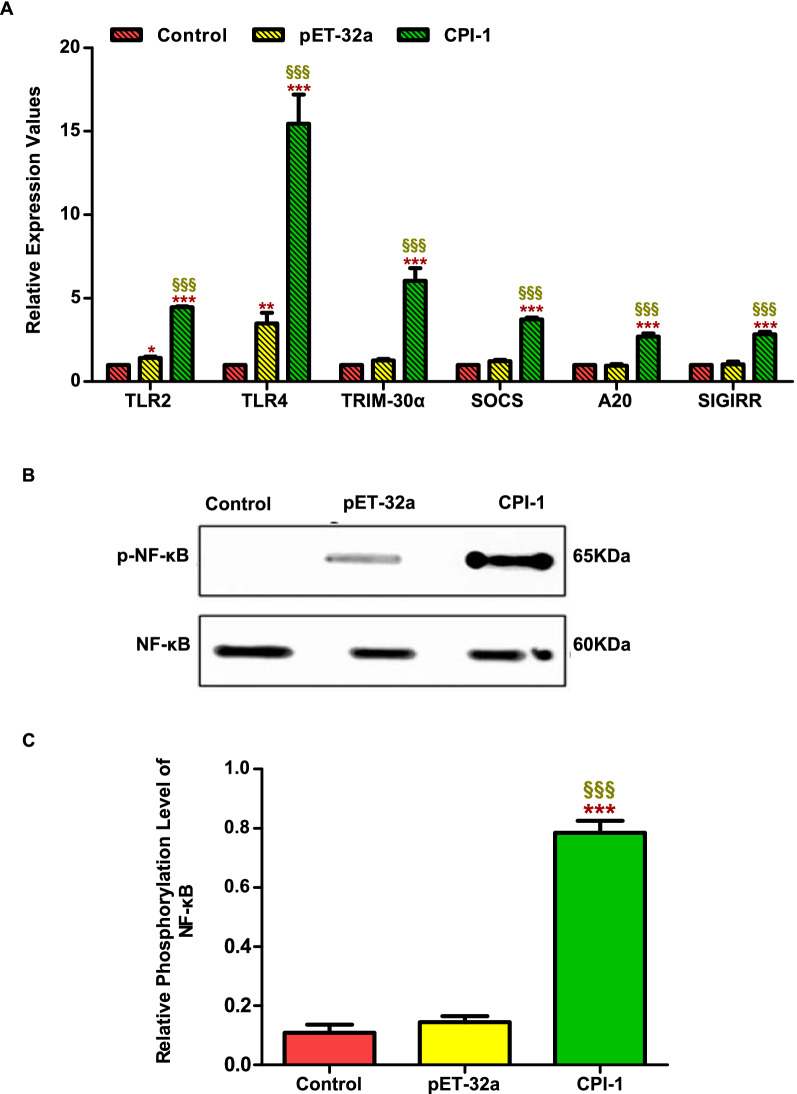
Analysis of the activation of the Toll-like receptor (TLR) signaling pathway in vivo. The relative expression of *Tlr2*, *Tlr4*, and the negative regulators of the TLR pathway are shown in **A**. A representative western blotting is shown in **B**, and the graph of the quantified band density is shown in **C**. Data are shown as mean ± SD of 3 replicates per group. ^*^P < 0.05, ^**^P < 0.01, ^***^P < 0.001 versus control group; ^§^*P* < 0.05, ^§§^*P* < 0.01, ^§§§^*P* < 0.001 versus pET-32a group

The in vivo experiments results showed that the transcription levels of TLR2, TLR4, TRIM-30α, A20, SIGIRR, and SOCS were significantly higher in the rBsCPI-1 group than those in the control and pET-32a group (*P* < 0.001; Fig. 10A). Western blotting showed that the phosphorylation level of NF-κB in the rBsCPI-1 group was significantly higher than that in the control and pET-32a group (Fig. 10B, C). In conclusion, rBsCPI-1 has a dual regulatory effect on the TLR signaling pathway activation and participates in regulating the phosphorylation of NF-κB signaling pathway.

## Discussion

The ability of parasites to resist the host immune response by releasing various immune regulatory factors signifies their precise regulation of the immune system [15–17]. Cysteine protease inhibitor (CPI) is a naturally occurring intracellular protease inhibitor widely expressed in all organisms ranging from protozoa to mammals [9, 10, 18]. CPI proteins from *Brugia malayi*, *Nippostrongylus brasiliensis*, and *Haemonchus contortus* have been shown to inhibit host cathepsin activity. Similarly, our previous studies have confirmed that rBsCPI-1, which is highly expressed in *Baylisascaris schroederi* migratory larvae, can effectively inhibit cathepsin L, cathepsin B, and papain in a dose-dependent manner (Xu et al.). Chen et al. [19] found that *Schistosoma japonicum* CPI impedes the ability of dendritic cells to present exogenous antigens. Dainichi et al. [13] proved that *N. brasiliensis* regulates the antigen processing pathway of host antigen-presenting cells by secreting CPI. Sun et al. [20] demonstrated that *Heligmosomoides polygyrus* CPI regulates the differentiation and activation of bone marrow-derived dendritic cells (BMDCs) and interferes with antigen processing. Therefore, we speculated that rBsCPI-1 may also have a certain regulatory effect on the function of immune cells.

PBMCs include various immune cells and play a critical role in innate and adaptive immunity [21]. Significant evidence indicates that PBMCs is a good model for studying the immune response and evaluating the efficacy of candidate vaccines, therapeutic drugs, and immunomodulatory molecules [21–23]. Giant panda species is greatly rare and precious, and previous studies have confirmed that mice infected with *B. schroederi* infectious eggs, the second-stage larvae (L2, migratory larvae) hatch in the small intestine and penetrate the intestinal wall, then migrate to the liver, lung, and other organs for parasitism [26], thus we chose mice PBMCs as the research object for the consideration of animal welfare. Pattern recognition receptors (PRRs), which mainly include TLRs and cytoplasmic NLRs, are a vital element of the innate immune system; they recognize pathogen-associated molecular patterns (PAMPs) as the first line of defense in monitoring pathogen infection [24, 25]. In the present study, ELISA assay results confirmed the presence of a significant interaction between rBsCPI-1 and PBMCs, and qRT-PCR further confirmed that rBsCPI-1 plays an immunoregulatory role mainly through the recognition of TLR2 and/or TLR4 on the surface of PBMCs. The activation of TLRs can be seen as a double-edged sword; it is essential for the host immune system to defend against parasitic infections, but the prolonged and excessive activation of TLRs leads to harmful inflammation and tissue damage in the host. Therefore, the TLR response needs to be strictly regulated [27, 28]. In this study, the transcription levels of SOCS, TOLLIP, SIGIRR, IRAK-M, IRAK-2, A20, and TRIM-30α, which negatively regulate the TLR signaling pathway, were also analyzed by qRT-PCR. The results showed that although rBsCPI-1 induced the activation of the TLR signaling pathway, it also limits its over-activation by enhancing the expression of the negative regulators.

PAMPs induce NF-κB activation by triggering PRRs, thereby promoting gene transcription and production of pro-inflammatory cytokines, and play a pivotal role in regulating the immune response to infection [29]. Mitogen-activated protein kinase (MAPK) cascade activation is the center of various signaling pathways and regulates cell proliferation-related signal pathways by receiving the signals converted and transmitted by membrane receptors and bringing them into the nucleus [30]. The MAPK family is divided into four subfamilies: ERK, p38 MAPK, JNK, and ERK5. Therefore, we analyzed the phosphorylation levels of NF-κB, ERK1/2, p38 MAPK, JNK1/2 by Western blotting. The results suggested that rBsCPI-1 transduces immune regulatory signals from extracellular to intracellular through the NF-κB and MAPK signaling pathways after being recognized by TLR2 and TLR4, thus affecting cell function and cytokines secretion.

Apoptosis plays a vital role in immune regulation and defense against infectious diseases. Recent studies have indicated that the induction of apoptosis upon parasite infection is caused by the interaction of parasite proteins with host cell proteins. The helminth-induced apoptosis of immune cells exhausts the host's immunity, paving the way for generating a permissive environment and chronic infection [31, 32]. Yu et al. [33] found that apoptosis occurred in the intestine of mice with *Trichinella spiralis* infection. Escamilla et al. [34] confirmed that *Fasciola hepatica* induced apoptosis in the peritoneal leucocytes of sheep in vivo. Zhou et al. [35] provided the first evidence that neuronal and astrocytic necroptosis and caspase-2-mediated apoptosis are induced by *Angiostrongylus cantonensis* infection in the parenchymal. In this study, we confirmed that rBsCPI-1 significantly induces cell apoptosis through FCM. Then, the transcription levels of pro-apoptotic genes *Bax* and *Fas* and anti-apoptotic genes *Bcl-2* and *Bcl-xl* were further determined using qRT-PCR; the results suggested that rBsCPI-1 has a dual regulatory effect mainly to promote apoptosis. Meanwhile, studies have shown that NF-κB has a dual regulatory effect on cell apoptosis [36, 37]. Similarly, activated MAPK also regulates cell apoptosis by transferring extracellular stimuli [38–40]. Western blotting results showed that rBsCPI-1 significantly activated NF-κB and MAPK. Therefore, we can preliminarily conclude that rBsCPI-1 triggers the apoptosis of PBMCs through the TLR/NF-κB and/or TLR/MAPK signaling pathway to inhibit the immune response combined with the results of apoptosis experiments.

The host eliminates apoptotic cells by enhancing cell phagocytosis, thereby inhibiting the release of possible pro-inflammatory and pro-immunogenic intracellular contents [41, 42]. The results of FCM indicate that rBsCPI-1 regulates the inflammatory response by inducing phagocytes to phagocytize apoptotic cells. Moreover, we found that anti-inflammatory mediators TGF-β, and IL-10 and pro-inflammatory mediators TNF-α and IL-1β were also significantly induced; however, the increased levels of anti-inflammatory factors were 2–4 times higher than pro-inflammatory factors. Therefore, rBsCPI-1 induces an anti-inflammatory immune response.

Chemokines play a crucial role in the host's defense and elimination of invading pathogens by inducing the directional chemotaxis of immune cells. Chemokines usually have four conserved cysteine residues [43, 44]. An exogenous cysteine protease inhibitor (such as rBsCPI-1) may increase the migration of immune cells by blocking the inhibitory effect of cysteine protease on the high expression of chemokines in the host. Furthermore, the release of chemokines is usually caused by the stimulation of inflammatory cytokines such as IL-1. Our results suggested that rBsCPI-1 stimulates the release of chemokines by inducing the expression of IL-1, thereby increasing the migration of PBMCs, which may facilitates the elimination of *B. schroederi*.

NO is widely distributed in various tissues and plays an essential role in immune regulation. NO is considered to be involved in most parasitic infections, mediating the host's non-specific defense by eliminating parasites or delaying parasite growth [45–48]. Consistent with previous studies [47, 49, 50], this study demonstrated that rBsCPI-1 significantly induces PBMCs to produce NO, which might promote the inflammatory response and pathogenesis during *B. schroederi* infection.

## Conclusions

By analyzing the effect of rBsCPI-1 on mice model through in vitro and in vivo experiments to get the following conclusions. On the one hand, rBsCPI-1 promotes the activation of the NF-κB and MAPK signaling pathways through TLR2 and/or TLR4 recognition and induces the production of pro-inflammatory factors and NO, thereby mediating an inflammatory response. On the other hand, rBsCPI-1 plays an immunosuppressive role by promoting the apoptosis of PBMCs, increasing their phagocytosis and inducing high expression of anti-inflammatory factors. Moreover, it also regulates the immune response to achieve the optimal state by inhibiting the excessive activation of the TLR signaling pathway. Thus, rBsCPI-1 has a dual regulatory effect on the immune system. The results of this study have essential significance for the protection of giant pandas and lay the foundation for studying the immune regulation mechanism of other ascarids.

## Supplementary Information

**Additional file 1: Table S1.** Primers for the investigated genes. The primer sequences in the table are used to detect the relative expression levels of the target genes in peripheral blood mononuclear cells (PBMCs) stimulated by PBS, pET-32a, and rBsCPI-1 by qRT-PCR.

**Additional file 2: Fig. S1.** Recombinant cysteine protease inhibitor of *Baylisascaris schroederi* migratory larvae (rBsCPI-1) affects the relative expression of Nod-like receptor (NLR) genes in peripheral blood mononuclear cells (PBMCs). Data are shown as mean ± SD of 3 replicates per group. ^*^ *P* < 0.05, ^**^ *P* < 0.01, ^***^ *P* < 0.001 versus control group.

## Data Availability

The datasets used or analysed during the current study are available from the corresponding author on reasonable request.
