# Supplementary material for: Regulatory effects of a novel cysteine protease inhibitor in Baylisascaris schroederi migratory larvae on mice immune cells
Source: Parasit Vectors. 2022 Apr 4;15:121. doi: 10.1186/s13071-022-05240-8 (PMC8981815; doi:10.1186/s13071-022-05240-8)
Supplement: Supplementary file 1 — Additional file 1: Table S1. Primers for the investigated genes. The primer sequences in the table are used to detect the relative expression levels of the target genes in peripheral blood mononuclear cells (PBMCs) stimulated by PBS, pET-32a, and rBsCPI-1 by qRT-PCR. [file 13071_2022_5240_MOESM1_ESM.doc]

**Table S1 Primers for the investigated genes**

|  | Primers | Sequence | Access Number |
| --- | --- | --- | --- |
| TLR1 | Forward | 5’-ACTCCTTAACTGACCTTCCT-3’ | AY009154.1 |
|  | Reverse | 5’-TCCACCACTTCTCTTGCTA-3’ |  |
| TLR2 | Forward | 5’-CCTTTTATTCTCTGGGCA-3’ | NM_011905.3 |
|  | Reverse | 5’-GTCAGGTGATGGATGTCG-3’ |  |
| TLR3 | Forward | 5’-GGATTGGTGAGTCTGAAGTA -3’ | AF355152.1 |
|  | Reverse | 5’-GGCTATGTTGTTGTTGCTTA-3’ |  |
| TLR4 | Forward | 5’-AACCAGCTGTATTCCCTC-3’ | NM_021297.3 |
|  | Reverse | 5’-TTTCTCACCCAGTCCTCA-3’ |  |
| TLR5 | Forward | 5’-CGAAGACTGCGATGAAGA-3’ | NM_016928.4 |
|  | Reverse | 5’-CTGGATGTTGGAGATATGGT-3’ |  |
| TLR6 | Forward | 5’-GGACTTAGAATACCTGGATGT-3’ | NM_001384171.1 |
|  | Reverse | 5’-GGAACTTGGCAGCACTTA-3’ |  |
| TLR7 | Forward | 5’-TGGACACGGAAGAGACAA-3’ | AY035889.1 |
|  | Reverse | 5’-CAGATGGTTCAGCCTACG-3’ |  |
| TLR8 | Forward | 5’-GAACATCACTCAGGAAGACT-3’ | AY035890.1 |
|  | Reverse | 5’-TCGGAACACATAGCCTCT-3’ |  |
| TLR9 | Forward | 5’-CAGCCACAACATTCTCAAG-3’ | NM_031178.2 |
|  | Reverse | 5’-CTCCAACAGTAAGTCTACGA-3’ |  |
| TLR11 | Forward | 5’-CATCTTCTCAACCAGTCAAC-3’ | NM_205819.3 |
|  | Reverse | 5’-ACCTCCTTCAATAGCATCTC-3’ |  |
| TLR12 | Forward | 5’-CTGTGTCTACTCTGCTTCC-3’ | NM_205823.2 |
|  | Reverse | 5’-TGCCTGCCTTCTTATCCA-3’ |  |
| TLR13 | Forward | 5’-GCCTTCACTCCTCTAATCAA-3’ | NM_205823.2 |
|  | Reverse | 5’-TCACCGTTCCACTCAGAT-3’ |  |
| NOD1 | Forward | 5’-GCTGAGATGCTGAGAGTG-3’ | AY160222.1 |
|  | Reverse | 5’-CTGTTATGGCTGTGTTCTTC-3’ |  |
| NOD2 | Forward | 5’-CCTTCTTGACCATCCTGAC-3’ | NM_145857.2 |
|  | Reverse | 5’-CTCTGTGCGAACGAACTT-3’ |  |
| NLRP1 | Forward | 5’-ACATCCACATACTGCTCAC-3’ | NM_001004142.2 |
|  | Reverse | 5’-ATCTTCACACCACCATCAC-3’ |  |
| NLRP2 | Forward | 5’-TAACCACACGGACACAGT-3’ | NM_177690.3 |
|  | Reverse | 5’-CCAGACAGAGGCAGAATATC-3’ |  |
| NLRP3 | Forward | 5’-AGACCTCCAAGACCACTAC-3’ | BC116175.1 |
|  | Reverse | 5’-ACATAGCAGCGAAGAACTC-3’ |  |
| NLRP4 | Forward | 5’-GCTCAGTCCTCAGAACCT-3’ | NM_001004194.2 |
|  | Reverse | 5’-GCCACCAACTTCATCTCTT-3’ |  |
| NLRP5 | Forward | 5’-CGAGTAGAGAAGTATGTTACCT-3’ | BC053384.1 |
|  | Reverse | 5’-ATGGATGTGTCAAGCAGAG-3’ |  |
| NLRP6 | Forward | 5’-GGTGAAGGAGAGGAATGC-3’ | NM_133946.2 |
|  | Reverse | 5’-GGATGAACAGTAGGCGATT-3’ |  |
| NLRP12 | Forward | 5’-TTACACTCGGCTTCTCCTA-3’ | NM_001033431.1 |
|  | Reverse | 5’-GTTCTTCGTCTGGCTCAA-3’ |  |
| A20 | Forward | 5’-TATGCCACGAATGCTCAG-3’ | U19463.1 |
|  | Reverse | 5’-CAACGCTCACAGAATCCA-3’ |  |
| IRAK-M | Forward | 5’-TGCCAGAAGAATACATCAGA-3’ | AJ440757.2 |
|  | Reverse | 5’-CAAGAACAGTGGAGAAGGA-3’ |  |
| IRAK-2 | Forward | 5’-CCTCCTCTCCACTCTTCA-3’ | BC085324.1 |
|  | Reverse | 5’-AAGTCTCTGTAGCGTTCTG-3’ |  |
| SOCS | Forward | 5’-AGAGGAAGTGACAGAGGAG-3’ | NM_009895.4 |
|  | Reverse | 5’-ACAAGGCTGACCACATCT-3’ |  |
| Tollip | Forward | 5’-GTGGAGGACGAGTGGTAT-3’ | BC062139.1 |
|  | Reverse | 5’-TTCAAGCACAGAACGGATT-3’ |  |
| SIGIRR | Forward | 5’-CAGAGATTGTGTCCAGTGT-3’ | BC094069.1 |
|  | Reverse | 5’-CGTAGGCATCGTATAACTTC-3’ |  |
| TRIM-30α | Forward | 5’-GAAGTAACCTGTCCTATCTGT-3’ | NM_001357467.1 |
|  | Reverse | 5’-TCATCCTGCCATTCATCAC-3’ |  |
| Bax | Forward | 5’-CTGATGGCAACTTCAACTG-3’ | NM_007527.3 |
|  | Reverse | 5’-ATCTTCTTCCAGATGGTGAG-3’ |  |
| Fas | Forward | 5’-AATCGCCTATGGTTGTTGA-3’ | BC061160.1 |
|  | Reverse | 5’-TGTGTCTTGGATGCTGTC-3’ |  |
| Bcl-2 | Forward | 5’-TCGTGACTTCGCAGAGAT-3’ | NM_009741.5 |
|  | Reverse | 5’-CAGAGACAGCCAGGAGAA-3’ |  |
| Bcl-xL | Forward | 5’-GGAGAGCGTTCAGTGATC-3’ | L35049.1 |
|  | Reverse | 5’-AGGTGGTCATTCAGATAGGT-3’ |  |
| TGF-β | Forward | 5’-GCAACAACGCCATCTATG-3’ | NM_011577.2 |
|  | Reverse | 5’-CAAGGTAACGCCAGGAAT-3’ |  |
| IL-10 | Forward | 5’-ACTGCTAACCGACTCCTT-3’ | NM_010548.2 |
|  | Reverse | 5’-TCCACTGCCTTGCTCTTA-3’ |  |
| TNF-α | Forward | 5’-GTGGAACTGGCAGAAGAG-3’ | NM_013693.3 |
|  | Reverse | 5’-GCTACAGGCTTGTCACTC-3’ |  |
| IL-1 | Forward | 5’-CTTCAGGCAGGCAGTATC-3’ | NM_008361.4 |
|  | Reverse | 5’-CAGCAGGTTATCATCATCATC-3’ |  |
| CCL2 | Forward | 5’-CAATGAGTAGGCTGGAGAG-3’ | NM_011333.3 |
|  | Reverse | 5’-GAAGTGCTTGAGGTGGTT-3’ |  |
| CXCL10 | Forward | 5’-GCAACTGCATCCATATCG-3’ | NM_021274.2 |
|  | Reverse | 5’-GACATCTCTGCTCATCATTC-3’ |  |
| GAPDH | Forward | 5’-TCTCCTGCGACTTCAACA-3’ | AY618199.1 |
|  | Reverse | 5’-TGTAGCCGTATTCATTGTCA-3’ |  |
