# Supplementary figures and images for: Regulatory effects of a novel cysteine protease inhibitor in Baylisascaris schroederi migratory larvae on mice immune cells
Source: Parasit Vectors. 2022 Apr 4;15:121. doi: 10.1186/s13071-022-05240-8 (PMC8981815; doi:10.1186/s13071-022-05240-8)

Relative Expression Values

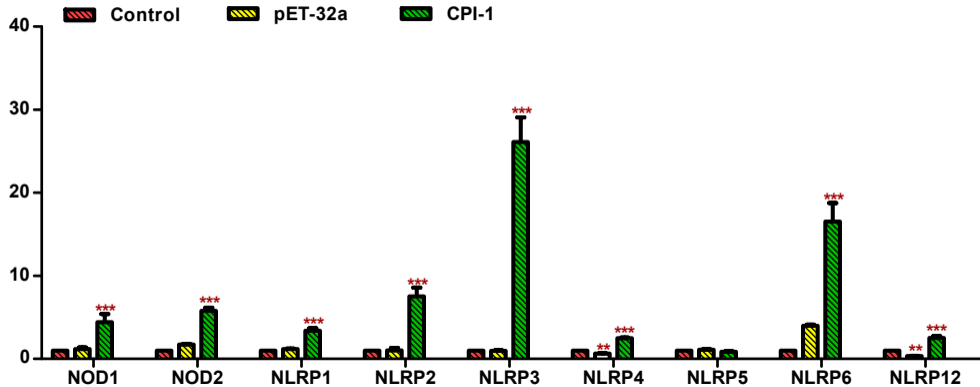

Supplement: Supplementary file 2 — Additional file 2: Fig. S1. Recombinant cysteine protease inhibitor of Baylisascaris schroederi migratory larvae (rBsCPI-1) affects the relative expression of Nod-like receptor (NLR) genes in peripheral blood mononuclear cells (PBMCs). Data are shown as mean ± SD of 3 replicates per group. * P < 0.05, ** P < 0.01, *** P < 0.001 versus control group. [file 13071_2022_5240_MOESM2_ESM.pdf]
